# The gap in dementia research: the need for translational research

**DOI:** 10.1186/1472-6963-14-S2-P104

**Published:** 2014-07-07

**Authors:** Martina Roes, Franziska Laporte-Uribe, Quasdorf Tina

**Affiliations:** 1 German Center for Neurodegenerative Diseases (DZNE), Witten, Germany

## 

Methods of translational research, implementation & dissemination, and its theoretical references, concepts of knowledge circulation, and criteria for assessment of these processes are established subjects of implementation and dissemination science. Due to the working group, ‘Implementation and Dissemination Research’ (ImDi) at the German Center for Neurodegenerative Diseases (DZNE) and the ‘Section Dissemination and Implementation (SDI)’ in the German Society of Nursing Science (DGP), the national and international discourses of the translational research has been more deeply thematized.

There are three different perspectives:

The quality of living with dementia depends on the person her/himself and her/his biography, the personal care and the social and material environment. Therefore, dementia care is culture specific. Experiences from other countries cannot always be transferred to the German context and particularly migrants need an extra awareness. Moreover the regulatory system is also important since the care potential depends on health care services and the whole health care system including reimbursement regulations (e.g. Long-Term Care insurance, LTCI).

From the perspective of a clinician it can be determined that translational research mainly focuses on the connection of fundamental and clinical research (e.g. drug development, longitudinal studies on biomarkers etc.).

From the perspective of implementation and dissemination research it can be determined that a) only a fraction of research results for people with dementia is translated into care practice; b) the implementation of care interventions is not carried out systematically; c) the systematic, structured, and sustainable implementation as well as the continuous evaluation of implementation effects are usually omitted, and d) research that focuses implementation processes is usually not funded. With this in mind, it can be concluded, that implementation and dissemination research in Germany represents a huge research gap.

